# Neural Hyperexcitability in Autism Spectrum Disorders

**DOI:** 10.3390/brainsci7100129

**Published:** 2017-10-13

**Authors:** Yukari Takarae, John Sweeney

**Affiliations:** 1 Department of Psychiatry, University of Texas Southwestern, Dallas, TX 75390, USA; 2 Department of Psychiatry and Behavioral Neuroscience, University of Cincinnati, Cincinnati, OH 45220, USA; sweenej5@ucmail.uc.edu

**Keywords:** autism, sensory abnormalities, inhibition, individual differences, sensory hypersensitivity, sensory hyposensitivity

## Abstract

Despite the progress that has been made in research on autism spectrum disorders (ASD), the understanding of the biological basis of ASD to identify targets for novel, effective treatment remains limited. One of the leading biological theories of autism is a model of cortical hyperexcitability. While numerous genetic and epigenetic studies support this model, how this particular biological alteration relates to known phenotypes in ASD is not well established. Using examples of sensory processing alterations, this review illustrates how cortical excitability may affect neural processes to result eventually in some core clinical phenotypes in ASD. Applications of the cortical excitability model for translational research and drug development are also discussed.

## 1. Introduction

Frequent seizures and sensory hyperreactivity in autism spectrum disorders (ASD) suggest cortical hyperexcitability in this population. Accordingly, an elevation in cortical excitability has been proposed to be a fundamental neurobiological characteristic for many patients with ASD [1,2]. While the model of cortical excitability has gained widespread support from genetic and epigenetic studies, its specific effects on observed behavioral phenotypes in ASD are not well established. This review will use examples of sensory processing abnormalities to illustrate how cortical excitability can influence neuronal processes eventually to affect behavioral phenotypes. Changes in cortical excitability, as seen in many animal models and clinical disorders, produce profound effects on sensory functions. This is because sensory systems rely on a dynamic, yet precise, balance of excitatory and inhibitory drives to discriminate and code features of incoming signals. In turn, sensory assessment can be a powerful tool for identifying changes in cortical excitability in clinical research and practice. We will first review evidence that supports elevation in cortical excitability in ASD, then discuss how increased cortical excitability can influence sensory processes via various neural processes. Finally, we will discuss therapeutic and translational applications of the cortical excitability model.

## 2. Evidence Supporting the Cortical Excitability Model in ASD

Several lines of genetic and epigenetic evidence suggest an elevation in cortical excitability in ASD by documenting gamma-aminobutyric acid (GABA) [3,4,5,6,7,8,9] and glutamate [10,11,12,13] system alterations. First, one of the most commonly observed copy number variations (CNV) in ASD is located on the chromosome 15q11 to q13 region, which contains several genes coding for subunit variants for GABA receptors [4]. Deletions involving these regions have been associated with Angelman or Prader–Willi syndromes, depending on the parental origin of the gene, and these syndromes are known for high incidence of ASD [8]. Duplications in the 15q11–13 region have also been observed by several studies with ASD [14,15,16]. The duplication of genes in this region leads to decreases, not increases, in GABA signaling, and are associated with epilepsy [8,17,18].

Second, the reduced expression of GABA-related genes, such as glutamic acid decarboxylase (GAD) 65 and GAD67 proteins has been observed in the cerebellum and parietal cortex in histological studies of ASD [5]. In addition, lower counts of GABAergic neurons, including Purkinje [19,20] and parvalbumin (PV) positive cells [21] have been reported in ASD. Further, reduced binding at GABA receptors is observed in the hippocampus [3] and anterior and posterior cingulate cortex [6,22]. These differences in gene expression and microstructures indicate a reduced GABA signaling in ASD. Third, increased glutamate receptors and glutamate transfer proteins have been additionally reported in ASD to further support the bias toward excitation. The mRNA levels of glutamate-system-related genes, such as α-amino-3-hydroxy-5-methyl-4-isoxazolepropionic acid (AMPA) 1 and amino acid transporter 1, are elevated in ASD [23]. Histone modification changes have also been associated with a glutamate receptor gene, glutamate ionotropic receptor NMDA type subunit 2A (GRIN2A), to indicate alterations in the glutamate pathways in ASD [13].

Fourth, macrolevel analyses using magnetic resonance spectroscopy (MRS) are also consistent with elevated excitability in ASD. Lower GABA concentrations have been reported in the motor [24], somatosensory [25], visual [26], and auditory [24,27] cortices. Increases in glutamate concentrations have also been reported in the auditory cortex [28] and occipital [26] areas. Reduced inhibitory effects or paradoxical facilitation following transcranial magnetic stimulation (TMS) have been observed in ASD participants [29,30], which corroborate the findings of neurotransmitter imbalance and the resulting bias toward excitation.

## 3. Effects of Elevated Excitability on Neurophysiological Function in ASD

### 3.1. Neural Synchronization

While there is increasingly robust evidence for an elevation in cortical excitability in ASD, cortical excitability has many mechanisms to affect cortical networks, via changes in synaptic formation, neural plasticity, and neural communication [21,31,32]. Thus, the influence of cortical excitability on cognition and perception is multifaceted, and its impact may vary across brain regions, in different behavioral conditions, and across different individuals. 

One of the best-documented impacts of increased cortical excitability is on neural communication within brain networks. Control of excitability affects neural activity in both phasic and tonic fashions [32,33]. The fast phasic form of control results from synaptically mediated effects at fast-spiking GABAergic neurons, and resulting inhibitory postsynaptic currents have a very rapid onset that allows for immediate effects on postsynaptic activation [32,34,35]. This rapid phasic influence enables precise control for neural activity timing, and thus is essential in coordinating neural signals to produce rhythmic, synchronized oscillations [36,37]. Synchronized oscillation is critical in coordinating the timing of interactions between neurons, which is essential to optimal neural communication [38,39].

Past electroencephalogram (EEG) studies have shown reduced neural oscillation in ASD [40,41,42], which suggests reduced phasic control. In particular, reduction in gamma oscillation during auditory and linguistic stimulus processing has been replicated in ASD by several groups [43,44,45]. Abnormal gamma oscillation is associated with impairments in language functioning in ASD [46] and is also seen in first-degree relatives [47,48]. These findings indicate that reduction in gamma oscillation is likely a shared neurophysiological feature for the population with hereditary components.

Reduced temporal control over neural activity also predicts more variable neural response timing across trials. Many neurons need to fire in synchrony to produce large enough physiological signal changes to be detected by techniques such as EEG and magnetic resonance imaging (MRI). Reduced neural synchrony results in more variable responses when the system is repeatedly stimulated. ASD participants are known to have greater intertrial variability in Blood-oxygen-level dependent (BOLD) responses during MRI sensory studies, and this is not accounted for by general signal-to-noise ratio differences [49,50]. Similar increases in the intertrial variability in ASD have also been documented in visually evoked responses using EEG techniques [51].

Reduced temporal control over the timing of neural activity is also likely to have effects on behavioral phenotypes in ASD. Because conscious perception of stimuli depends on the duration of cortical sensory responses [52,53], precise control for the onset and offset of the neural response is essential for an accurate perception of stimulus durations. Consistent with this idea, studies of typically developing (TD) adults have shown that the ability to differentiate temporal characteristics of stimuli decreases as levels of cortical excitability increase [54,55]. Hence, lower discrimination ability for temporal information is expected in populations with elevated cortical excitability, such as ASD. This is consistent with impairments in the detection and discrimination of subsecond or fast oscillating stimuli that have been reported in ASD [25,56,57]. These impairments in processing temporal aspects of stimuli appear to be more severe in individuals with ASD with clinically evident sensory hypersensitivity [56] or lower GABA levels in the relevant sensory cortex [25].

### 3.2. Neural Response Scaling

In addition to the fast phasic influence, cortical excitability can affect gain control of pyramidal cell activity in a more tonic fashion. This occurs through the generation of inhibitory currents via drive from extrasynaptic GABA receptors, which are located on soma, dendrites, and axons that are distant from synaptic neurotransmitter release sites [31,32]. These inhibitory currents affect levels of resting neuronal depolarization, and thus modulate neuronal readiness to produce action potentials [32,33]. Hence, tonic excitability control affects the likelihood of local neural communication and the magnitude of response, rather than the precise timing of neural communication. This effect is typically observed as a change in scaling of response magnitude per stimulation (i.e., response gain), which results in an enhanced response at middle- to high-intensity stimulation and reduced response saturation [33]. Drugs that affect neuronal excitability are known to produce similar disproportionate changes at middle- to high-intensity stimulation [58,59,60]. Consistently, some populations with known cortical hyperexcitability, such as epilepsy, show abnormal regulation of neural system output that is characterized by disproportionate scaling and reduced response saturation [61,62].

**Figure 1:**
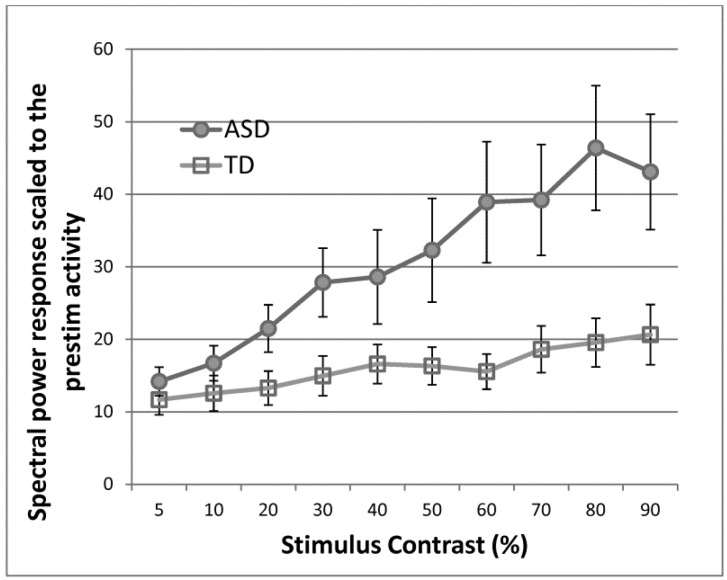
A greater increase in electroencephalogram (EEG) spectral power at the frequency of stimulus oscillation (monochromatic sinewave gratings) over varying levels of visual stimulus contrast in autism spectrum disorders (ASD) and typically developing (TD) groups, indicating greater cortical reactivity in ASD. From Takarae et al. 2016 [63], with permission.

We have shown that abnormal response scaling under visual stimulus contrast manipulation is present in ASD participants who are seizure-free [63]. We measured steady-state visually evoked responses with a wide range of stimulus contrasts in participants with ASD and age- and IQ-matched TD control participants (Figure 1). The spectral response at the stimulus frequency was greater in ASD than in control participants. More importantly, ASD participants showed a greater rate of response increase as the stimulus contrast increased, which is similar to the pattern observed in epilepsy patients.

Additional evidence indicating the difficulty in regulating neural output includes enhanced activation in the visual cortex in functional MRI (fMRI) studies, where suprathreshold, high-contrast stimuli were used [64,65]. Enhanced visual responses are considered as one of the most common fMRI findings in ASD [65]. These reports of heightened neural responses to sensory events may relate to clinical reports of sensory hypersensitivity or overresponsivity in everyday situations.

Psychophysical studies in ASD are consistent with a disproportionately elevated neural scaling when a wide range of suprathreshold stimuli are used. Greater sensitivity to middle- to high-intensity stimuli has been documented by lower thresholds for blink startle responses to loud sounds [66,67] and compressed auditory dynamic range, where stimuli were judged as being uncomfortable at a lower intensity [68]. Visual motion perception is also enhanced under high-contrast conditions in ASD [69].

### 3.3. Neural Habituation and Adaptation

Problems with the dysregulation of neural response amplitude may be exacerbated when the sensory system is repeatedly stimulated. Repeated stimulation typically results in habituation. Habituation, or repetition suppression, refers to a reduction of neural activity when stimuli are repeated. This is an important neural mechanism to differentiate “old” and “new” stimuli to code history with the particular stimulus. ASD individuals are known to show less neural habituation [70], or even increased responses [71], to repeated stimuli. This trait for the reduced ability for habituation may be familial [72] and is also seen in a related genetic condition, Fragile X [73].

Although habituation is a complex phenomenon that is probably related to both perceptual and postperceptual processes, reduced habituation could contribute to both hyper- and hyposensitivity to sensory events that are observed in ASD [72]. Reduced habituation results in smaller neural signal differences between new and old stimuli. Thus, old repeated stimuli do not earn an “old” status and continue to evoke relatively high responses like “new” stimuli, which may lead to more exaggerated behavioral responses. At the same time, this could result in the failure to assign a “new” status to new stimuli, which could lead to failure in executing appropriate orienting or other exploratory behaviors that are typically performed with new stimuli. This lack of orienting and exploratory behaviors to new stimuli may be regarded as sensory hyposensitivity.

ASD individuals also show reduced adaptation [64,74,75,76]. Habituation and adaptation are related concepts, and both are a part of activity-dependent neural plasticity to adjust neural responses dynamically for the optimal processing of sensory information [77,78,79]. While habituation paradigms typically examine response suppression to a single class of repeating stimuli, adaptation paradigms typically investigate changes in response selectivity of the system after extensive exposure to stimuli. The response property changes because extensive exposure could change the balance between sets of neurons with opposing response selectivity. Sensory neurons with different response selectivity are known to inhibit each other and also to receive inhibitory input from each other; inhibitory influence over other neurons is a function of the activity level of the neuron. This process of mutual inhibition, called opponency, instantiates inhibitory interactions within local circuitries. Opponency is especially critical in determining how much influence single neurons have in the population response because each neuron’s signal is weighted by both the relevant activation and inhibition processes [80,81]. Thus, the disruption of opponency by sensory adaptation could change how the sensory system responds to incoming signals.

Sensory adaptation has been well studied in the context of visual motion perception, and we have shown that sensory adaptation to visual motion is reduced in ASD [64]. In a typical experiment to induce visual motion adaptation, participants view directional movement for a prolonged period. After prolonged viewing, visual neurons that are sensitive to the movement direction experience response saturation. As a result, both their activity and their ability to maintain inhibitory influence over other neurons with opposite direction tuning decrease [82,83]. This temporary disruption in inhibitory interaction results in a relative increase in activation, via disinhibition, in the neurons with opposing direction tuning. This relative disinhibition yields the illusory perception of movement in the opposite direction (motion aftereffect, also called the waterfall illusion) [82,84]. Time course of activity in V5, extrastriate areas known to be sensitive to visual movement, is critically related to the motion aftereffect [85,86]. Decay of the V5 BOLD signal after motion adaptation is related to dissipation of the disinhibition resulting from the temporal disruption of inhibitory interactions (thus resumption of mutual inhibition) [85,86]. This V5 BOLD response after motion adaptation decays faster in ASD individuals [64]. Because the magnitude of disinhibition depends on the strength of the existing inhibitory interactions, a faster recovery suggests reduced inhibitory interactions. Consistent with these concepts, lower GABA levels obtained from MRS are associated with an increased magnitude and a shorter duration of BOLD responses in TD adults after viewing visual stimuli [87,88].

Similar principles based on disruption in mutual inhibition apply to adaptation to other stimulus features, such as orientation [89,90,91], and even higher-level stimuli, such as faces [92]. Laboratory studies with tactile stimulation have reported reduced neural adaptation in both adults [74] and children [76] with ASD. ASD individuals are also known to have less adaptation to faces [75]. Adaptation decreases neural responses to the adapted stimulus, while increasing responses to one that is orthogonal to the adapted stimuli. Thus, adaptation distorts the response property of the system and yields to temporary hyper- and hyposensitivity along the relevant stimulus dimension.

## 4. Individual Differences

**Figure 2:**
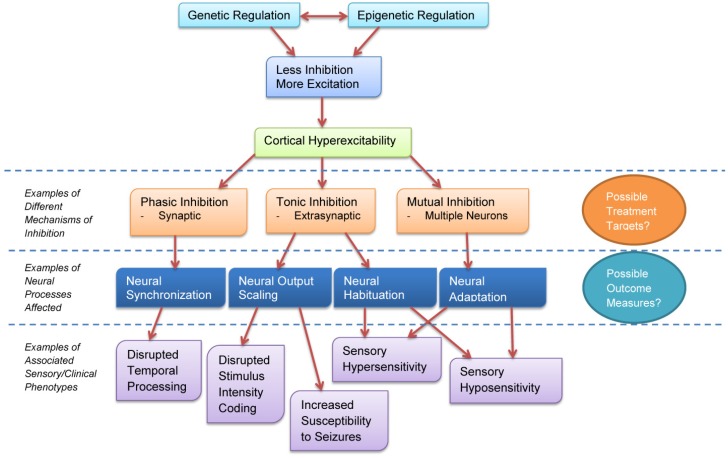
Cortical excitability affects neural inhibition through different mechanisms, each of which has cascading effects on sensory and clinical phenotypes in ASD.

We have discussed how cortical excitability influences neural synchronization, output scaling, habituation, and adaptation, and how these neural processes are altered in ASD. These neural changes are consistent with documented perceptual changes in ASD and are consistent with sensory abnormalities observed clinically. Figure 2 shows a schematic diagram of how these different neural changes affect behavioral phenotypes in ASD.

**Figure 3:**
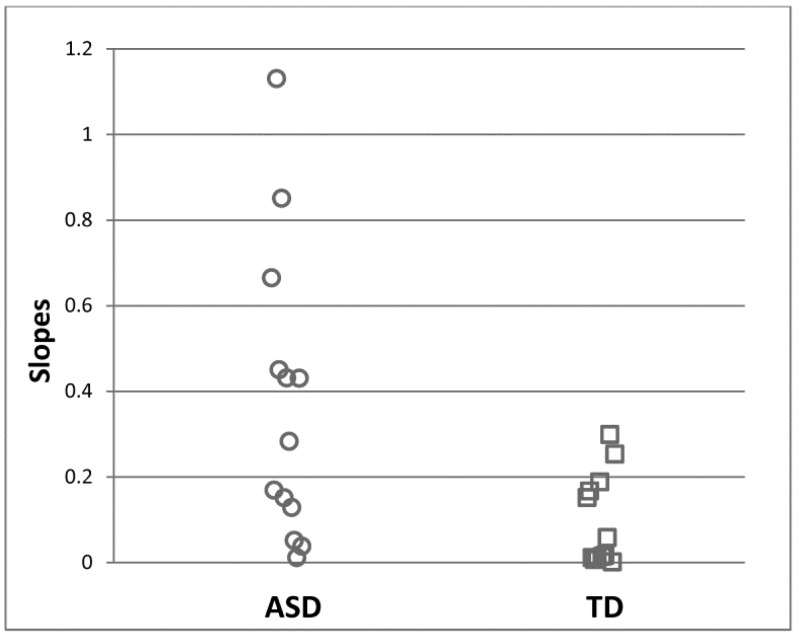
44% of ASD participants showed greater spectral power increases over contrast manipulation of the visual stimuli than any TD participant. Linear slopes for individual EEG responses across increasing visual contrast were estimated. From Takarae et al. 2016 [63], with permission.

It is worth noting that alterations in cortical excitability seem to exist to varying degrees in individuals with ASD, and only a subset of affected individuals may have neuronal hyperreactivity [29,30,63]. In the previously discussed steady-state study of visually evoked potentials [63], individual slopes for response increases to the visual contrast manipulation were quantified by fitting linear functions. The linear slope was greater in the ASD group than in the control group, indicating greater neural responsivity to visual stimuli at the group level. Here, 44% of ASD participants had slopes larger than any control participants (Figure 3), but the remaining participants had slopes that overlapped with the control group. This observation suggests that only a subgroup of individuals with ASD have neuronal hypersensitivity. Other studies also found that changes in cortical excitability were not universally observed in the participants. Post-TMS inhibitory effects were reduced in 37% of participants [30] and were more common in individuals with a history of early language delay [29]. Thus, there appears to be a fairly large range of cortical excitability in ASD, and perhaps only a specific subgroup with distinct clinical characteristics shows elevated cortical excitability. Thus, the ability to capture individual differences in cortical excitability may have a significant impact when cortical excitability is considered as a treatment target, as patients might be stratified before a treatment based on their pretreatment neurophysiological testing.

In addition to individual differences in overall levels of cortical excitability, further examining specific aspects of cortical excitability may clarify which potential neural mechanisms contribute to cortical hyperexcitability in ASD and possibly inform us about biological heterogeneity in the disorder. Different aspects of excitability control can result from distinct genetic and epigenetic factors, as well as atypical developmental trajectories [31,32]. For example, phasic inhibition is critical for neural synchronization and is primarily associated with GAD65, which is predominantly expressed in axons [93]. Tonic inhibition is critical in regulating neural output and is associated with GAD67, which is primarily expressed in soma [93]. Further, GAD65 and GAD67 have different regional distributions and developmental trajectories, suggesting that they might have distinctive effects on brain systems [94,95]. Thus, differentiating between specific effects of cortical excitability could provide new insights into the pathophysiology and possible pharmacological targets for individuals with ASD.

## 5. Cross-Species Comparisons

It has been shown that many preclinical models of ASD have abnormal sensory processing and altered balances for excitation and inhibition. The similar elevation of cortical excitability to the human data warrants well-controlled cross-species studies using identical tasks to seek parallel neurophysiological characteristics, such as changes in neural synchronization and neural output scaling. In particular, EEG and event related potentials (ERP) provide excellent tools for the investigation because homologues of human ERPs and oscillatory activity have been identified in rodents. This provides a particularly strong framework to link findings from rodent models to studies of human diseases and to develop novel translational research programs. ERP and oscillatory activity have a high reliability, which is suited for such investigations [96,97,98,99]. The framework may also be useful for investigating the molecular basis for specific symptoms in syndromic forms of ASD. For example, both human and rodent forms of Fragile X show sensory hypersensitivity [100,101]. Increased amplitude of ERP components, such as N1 and P2, are observed in both human participants and the fmr1 knock out mouse model of Fragile X [101]. The abnormally large ERP response becomes normalized by GABA agonists [102]. The enhanced neural and sensory responses are also consistent with the findings from slice physiology that show decreases in PV-positive inhibitory neuron activity in Fragile X [103]. The cross-species homologue at multiple levels of analysis brings opportunities to address cellular and circuit level abnormalities in the disorder to facilitate greatly the development of pharmacological targets.

## 6. Conclusions

Elevation in cortical excitability is observed in ASD at genetic, epigenetic, neural, and behavioral levels. While changes in cortical excitability affect general cognitive function, cortical excitability has especially profound effects on sensory phenotypes in ASD. The effect on sensory phenotypes is complex, affecting sensory processes through multiple different neural processes, which may contribute to the heterogeneous expression of symptoms. Despite the complex effect, close homologues in human and animal models offer great promise for developing models of cortical excitability and their neural mechanisms and creating novel therapeutic strategies. When combined with the ability to capture individual differences, these measures of cortical excitability have high potential for predicting and evaluating therapeutic outcomes to such therapies.
